# Novel techniques for intraoperative assessment of margin involvement

**DOI:** 10.3332/ecancer.2018.795

**Published:** 2018-01-10

**Authors:** Dorin Dumitru, Michael Douek, John R Benson

**Affiliations:** 1 The Royal Hampshire Hospitals, NHS Foundation Trust, Winchester SO22 5DG, UK; 2 Cambridge University Hospitals, NHS Foundation Trust, Cambridge CB2 0QQ, UK; 3 Division of Cancer Studies, King’s College, London WC2R 2LS, UK; 4 Guy’s and St Thomas’ Hospitals, NHS Foundation Trust, London SE1 9RT, UK; 5 School of medicine, Anglia Ruskin University, Cambridge CB1 1PT, UK

**Keywords:** breast surgery, margin assessment, technologies, re-excision, recurrence

## Abstract

Breast conserving surgery (BCS) is now the standard of care for the majority of women with early stage breast cancer. There is a finite rate of ipsilateral breast tumour recurrence (IBTR) for breast conserving therapy (BCT) with annual rates of less than 1% for specialist breast practices.

There has been recent consensus on the definition of an adequate resection margin for both invasive and noninvasive breast cancer treated with BCS, although some variation in margin policy persists with definitions of ‘no tumour at ink’, 1 and 2 mm margin mandates.

Despite the development of methods for intraoperative assessment of margins, up to 20% of patients require further surgery (cavity re-excision or completion mastectomy) to achieve clear surgical margins.

In the past decade, several novel technologies for intraoperative margin assessment have been explored with the aim of reducing rates of re-operation and its attendant patient anxiety, inconvenience and additional cost. Ongoing studies are addressing the safety, feasibility and cost-effectiveness of these novel technologies relative to methods in routine clinical usage.

## Introduction

Breast conserving surgery (BCS) has become the preferred standard of care for surgical management of women with early stage breast cancer over the past 30 years (NIH Consensus Conference 1991). Successful BCS entails excision of the tumour with an adequate amount of surrounding normal breast parenchyma, such that negative resection margins are obtained. Longer term follow-up data from several prospective randomised controlled trials have demonstrated survival equivalence for BCS combined with breast radiotherapy, when compared with *radical* or *modified radical* mastectomy [1–4]. Introduction of breast conserving therapy (BCT) has coincided with instigation of widespread mammographic screening, smaller tumour size at presentation and improvements in adjuvant therapies (radiotherapy, chemotherapy and hormonal/biological therapies). There is a finite rate of ipsilateral breast tumour recurrence (IBTR) for patients undergoing BCT, with current estimates supporting an annual rate of less than 1% for specialist breast practices and corresponding rates of between 3.5% and 6.5% at ten years [5]. Systemic chemo-hormonal therapies reduce rates of IBTR by approximately one-third, whilst anti-HER2 therapies can lead to halving of local in-breast recurrence.

Despite these dramatic improvements in rates of local control following BCT, between 20% and 30% of patients with either invasive or noninvasive disease typically require re-operation following initial BCS (be this cavity re-excision or mastectomy). These high rates of re-excision are often prompted by a component of ductal carcinoma *in situ* (DCIS), which usually has no clinical correlate and may be underestimated in its extent radiologically.

## Definition of negative margins

**Figure 1.**
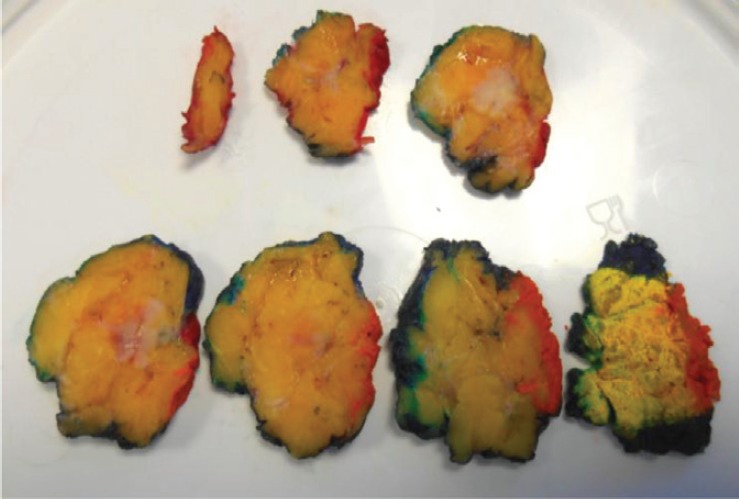
Macroscopic assessment and inking breast wide local excision specimen.

Until recently, there has been much variation in opinion amongst surgeons and radiation oncologists as to what constitutes an adequate or ‘negative’ surgical margin after BCS. Surgical margin status is considered as a major predictor of IBTR, but no consensus on what constitutes an ‘*adequate*’ width of surgical margin existed until the dual publication of an international consensus statement in 2014 [6]. Breast conservation surgery represents a balance between oncological mandates and cosmetic outcomes with surgeons aiming to excise tumour with ‘*negative*’ margins and acceptable cosmesis; the closer ink is to tumour, the narrower are the margins and a positive margin is associated with ink on cancer cells (Figure 1). Singletary [7] published one of the first major reviews of surgical margins at the beginning of the millennium and concluded that it was unacceptable to have tumour at the margin, as positive margins were associated with a doubling of rates of IBTR compared with nonpositive margins. However, there was no correlation between the width of the surgical margin and rates of IBTR. A large meta-analysis of 21 studies involving 14,571 patients with invasive breast cancer was published by Houssami *et al* [8] in 2010. This confirmed that the odds ratio for local recurrence was 2.42 [p < 0.001] for positive margins (i.e., tumour at ink). Of note, there was no statistically significant difference in rates of local recurrence between individual margin widths of >1, >2 or >5 mm. In particular, a margin width of 2 or 5 mm was not necessarily better than a narrower margin of 1 mm when results were adjusted for follow-up time and receipt of a radiation therapy boost and endocrine therapy. A 1 mm margin was therefore deemed to be adequate, provided patients received optimal adjuvant therapy [8]. An updated meta-analysis by the same authors involving involving 28,162 patients with invasive breast cancer (IBTR = 1506) employed random-effects logistic meta-regression, defined as a relative category of ‘close’ with cancer cells between a defined distance (negative margin) and the surgical resection margins (no tumour at ink) [9]. The odds ratio for IBTR was 1.98 [p < 0.001] for positive or close margins compared with negative margins and 2.44 [p < 0.0001] for positive versus negative margins. There was no statistical evidence that increase in margin width from ‘no tumour at ink’ to 1, 2 or 5 mm influenced the odds of local recurrence with adjustment for follow-up time. Nonetheless, it should be noted that this meta-analysis contained a few cases permitting direct comparison of a 1 mm margin versus ‘no tumour at ink’. For this reason and based on the results of the initial meta-analysis [8], the Association of Breast Surgery in the United Kingdom have adopted 1 mm as the minimum margin mandate for invasive disease (with or without admixed DCIS) and also for pure forms of DCIS. By contrast, the international consensus statement emphasised that an adequate margin exists when tumour is not touching ink, and this has been widely adopted in the past couple of years by more than two-thirds of surgeons in the United States. Indeed, this minimisation of margin mandate has led to a reduction in rates of re-excision at the Memorial Sloan-Kettering Cancer Centre from 21% to 15% [10]. (Table 1)

## Intraoperative specimen imaging

**Figure 2.**
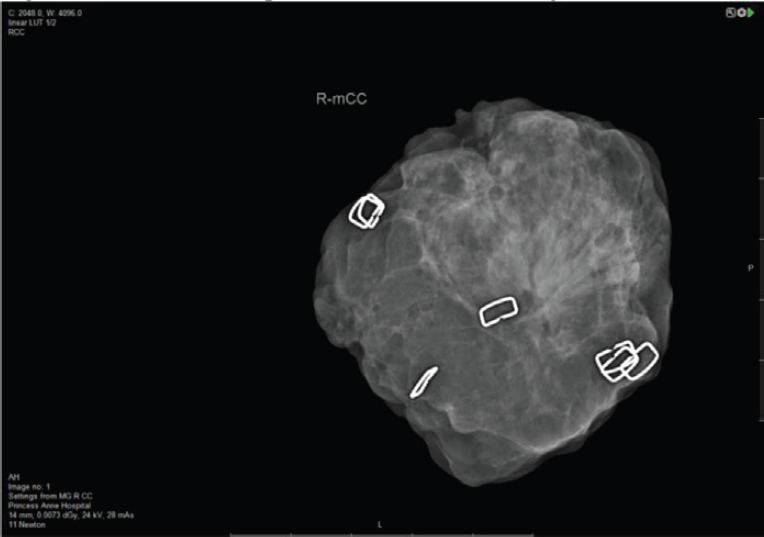
Faxitron: intraoperative assessment of margins in BCS.

Notwithstanding the above rates of re-excision and re-operation, following routine BCS for both palpable and impalpable lesions remains high and this has spurred efforts to develop reliable intraoperative assessment tools which can provide a timely indication of whether re-excision of a cavity margin is indicated at the time of primary surgery. More conventional imaging methods of specimen radiography and intraoperative ultrasound (IOUS) share the limitation of detecting DCIS and microscopic margin involvement, whilst pathological assessment with frozen section and touch imprint cytology is labour intensive. Many surgeons perform intraoperative radiological assessment of impalpable (and sometime palpable) lesions using portable X-ray devices such as the Faxitron; McCornick *et al* [11] found that use of intraoperative specimen radiography more than halved rates of positive margins (reduced from 12% to 5%) whilst Layfield *et al* [12] reported that use of intraoperative specimen radiography for palpable breast cancer led to a reduction in the mean specimen weight without increasing re-excision rates and was associated with improved cosmesis. In a prospective study involving 170 cases of both palpable and impalpable lesions, margin status was assessed systematically with intraoperative specimen radiography, using a dedicated intraoperative X-ray device (Faxitron MX20). Margins were evaluable in 91.2% of cases and were assessed according to the histological subtype; thus, attaining a 10 mm margin on Faxitron led to negative margins in 98.5% of invasive ductal carcinomas, 90% of invasive lobular carcinomas and 78.5% of DCIS cases with an overall margin positivity rate of 6.5% when intraoperative specimen radiography was employed. (Figure 2) The correspondingly high negative margin rate (93.5%) can potentially lead to savings of time and costs.

Another intraoperative imaging modality for reducing rates of margin positivity at initial surgery is IOUS, which can be performed by surgeons who are increasingly ultrasound competent. In a prospective observational cohort study, Karalik *et al* [13] undertook BCS guided by either ultrasound (n = 84) or palpation (n = 80) amongst two groups of women matched for demographic and tumour characteristics. Use of IOUS reduced the rate of re-excision from 17% for palpation-guided lumpectomy to only 6% with IOUS. (p = 0.03). Moreover, the volume of tissue resected in the palpation-guided group was significantly higher despite comparable tumour size (p = 0.048) and equivalent cosmetic outcomes.

The COBALT study is a prospective multicentre trial which addressed the hypothesis that use of IOUS for wide local excision of palpable breast cancers could potentially spare healthy tissue and improve both surgical margin status and cosmesis. The trial was conducted in the Netherlands, but accrued a relatively small number of patients in relation to the number of centres and participating surgeons. Notwithstanding this comment, the trial had 80% power to detect an 18% reduction in the re-excision rates. A total of 134 patients with palpable early stage primary invasive breast cancer (T1-2, N0-1) were randomised to either ultrasound-guided surgery (USS) or palpation-guided surgery (PGS). Some patients had invasive tumour associated with DCIS, which influenced rates of margin positivity. Nonetheless, use of ultrasound significantly increased the negative margin rate from 83% (PGS) to 94% (USS) (p = 0.03). Moreover, specimen volumes at first excision were significantly lower for the USS group compared with the PGS group (p = 0.048). The trial contained a quality-of-life sub-study and also examined cosmetic outcomes [14].

Another familiar imaging modality is MRI which can be used for specimen radiography; however, this methodology within the context of surgical excision specimens is impractical, time consuming and unlikely to be cost-effective in any managed healthcare system.

## Routine cavity shaves and intraoperative margin pathology

Several observational studies have shown a significant reduction in rates of re-excision when additional tissue is routinely removed from all margins of the surgical cavity (x 6) [15, 16]. However, some of these studies report adverse effects on cosmesis, and a report by Coopey *et al* [17] found that routine cavity shaves do not impact upon rates of re-excision and indeed the volume of tissue excised in the main specimen was less for the cavity shave group, suggesting a conscious modification of surgical technique when immediate cavity re-excision was anticipated. More recently, a randomised trial involving 235 patients with stage 0–III breast cancer undergoing BCS with or without routine cavity shaves was published in the New England Journal of Medicine [18]. Randomisation to routine cavity shaves led to a statistically significant reduction in the margin positivity rate (19% versus 34%; p = 0.01) compared with equivalent rates prior to randomisation (36% shave group; 34% non-shave group). This was associated with a commensurate reduction in rates of re-operation to achieve negative margins for the shave compared with non-shave groups (10% versus 21%), respectively, p = 0.02). Interestingly, these authors found no statistically significant difference in the total specimen weight between the two groups. A comparison of different intraoperative margin management techniques was reported by Bolger *et al* [19], who examined three approaches – routine cavity shave margins (n = 70), macroscopic assessment of margins (MMA) (n = 68) and no formal margin assessment (n = 50). Routine cavity shaves with a minimum thickness of 1 cm were taken from four surfaces and MMA involved the pathologist examining the specimen for assessment of margin status. The distance of tumour from each radial margin (medial, lateral, superior and inferior) was measured and if less than 5 mm, this prompted re-excision of that margin. The remaining group underwent re-excision at the discretion of the operating surgeon. Comparison of the two groups with formal assessment of margins revealed a re-excision rate of 25% compared with 34% for the group without formal evaluation of margins and a statistically significant reduction in the chance of residual disease following the initial surgical procedure (p = 0.02). Formal assessment did not lead to any notable prolongation of operating time.

Both frozen section and touch imprint cytology are time consuming and require input from a pathologist; in terms of diagnostic accuracy, frozen section was found on meta-analysis to have a pooled sensitivity of 0.86 (95% CI 0.78–0.91) and a specificity of 0.96 (95% CI 0.92–0.98), but with significant heterogeneity between studies. By comparison, cytology had a pooled sensitivity of 0.91 (95% CI 0.71–0.97) and a specificity of 0.95 (95% CI 0.90–0.98). Once again, there was significant heterogeneity between studies with an I2 exceeding 90% [20]. In terms of comparative performance, these tissue-based techniques are most accurate for intraoperative margin assessment, but have very poor uptake in routine clinical practice. This most likely relates to the logistical issues of slow turnaround times, disruption of operating lists and availability of pathology staff [20]. Use of frozen section in American practice is reported to reduce rates of re-operation from 13.2% to 3.6%, although it remains unclear what proportion of practices in the United States employs methods for intraoperative assessment of margins [20].

## Novel technologies for margin assessment

In recent years, several novel technologies have been explored for assessment of margin status in light of the limitations of the above methods, especially for impalpable lesions and DCIS. It is essential that these newer technologies have similar diagnostic accuracy to conventional methods involving pathological examination of tissue. However, they must also offer advantages in terms of turnover times, practicality and cost before introduction into routine clinical practice (especially in a managed healthcare system).

Some of these can detect microscopic malignancy at the edges of the surgical specimen and therefore have potential to reduce the margin positivity and rates of re-excision to a much greater degree than conventional methods for intraoperative specimen radiography. These newer methods for assessment of surgical margins generally involve some form of electromagnetic waves which can penetrate the surface of the specimen to a variable depth and can distinguish between benign and malignant tissue in real time. Interestingly, recent reduction in the margin mandate for both invasive and noninvasive cancer favours these emergent technologies which have limited tissue penetrance and are ideally suited for detection of cancer cells within 1 mm of the inked specimen margin [20].

## MarginProbe

**Figure 3.**
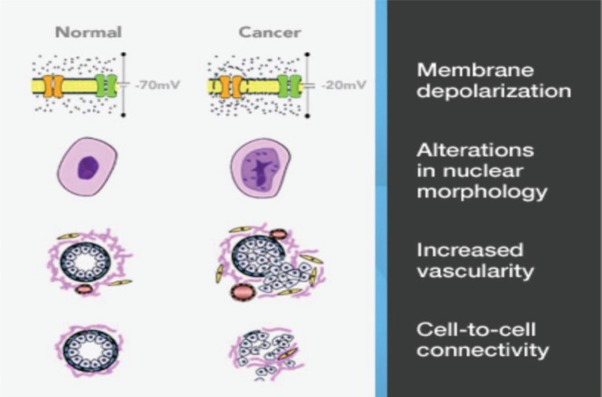
Marginprobe: tissue assessment.

One such technology is the MarginProbe device (Dune Medical Services Limited, Caesarea, Israel) based on reflection of radiofrequency waves that measure local electrical properties of breast tissue within the radiofrequency range. The device creates an electrical signature of tissues that is dependent on the membrane potential, nuclear morphology and intercellular contact. The device also takes account of differences in vascularity, and collectively these factors permit distinction between malignant and benign/normal tissue. (Figure 3) The overall sensitivity for detection of malignancy is 70–100%, but specificity is slightly lower at 70–87% [21]. Of note, these performance parameters are similar for invasive carcinoma (ductal and lobular) and DCIS [22]. The MarginProbe has been designed as an adjunct to routine surgical methods, the hand-held device is convenient to use and the probe has a 7 mm footprint which is employed to take between five and eight measurements from each of six surfaces of the excised specimen (previously defined with an ink pen). A positive reading indicates tumour cells present within 1 mm of the resection margin. Any positive reading is interpreted as a positive margin and re-excision of that margin is undertaken.

Early studies with MarginProbe randomised patients to routine surgical technique (including re-excision prompted by palpation or specimen radiograph) or usual technique combined with MarginProbe. These have revealed reductions in rates of re-excision from 25% to 12% for the device arm with no differences in the cosmetic outcomes [23]. The MarginProbe device can therefore increase the ability of surgeons to identify and excise positive margins at the time of surgery, without employing pathological methods for intraoperative assessment such as frozen section or cytology. In consequence, MarginProbe can reduce rates of re-operation for the benefit of patients and the healthcare system [24].

## ClearEdge™

Another related technology is the ClearEdge™ (CE) imaging device which measures tissue-specific electrical properties. It is also a convenient hand-held portable imaging device which uses bioimpedance spectroscopy to detect both extracellular and intracellular variations in tissue dielectric properties, enabling identification of abnormal tissue containing pathological changes of invasive cancer, DCIS, lobular carcinoma *in-situ* (LCIS) and atypical proliferations such as atypical ductal and lobular hyperplasia. The specificity is slightly compromised by detection of increased cellularity associated with inflammation. The device assesses the local dielectric properties of tissues when directly in contact. A preliminary study was done under the auspices of Michael Dixon in the Edinburgh Breast Unit, which enlisted nine surgeons and was conducted in two phases. Phase-1 (58 patients) was designed to validate the safety and accuracy of the CE imaging device when used on excised specimens *ex vivo*. This would indicate the potential for the device to reduce rates of re-excision, which was specifically addressed in Phase-2 of the study, whereby the device was used intraoperatively in 63 patients in an attempt to reduce the need for re-operation to achieve negative margins. The performance of the CE imaging device was similar for the two phases of the study and readouts from the device were compared with permanent section pathology (sensitivity [84.3% versus 87.3%]), specificity (81.9% versus 75.6%), positive predictive value (67.2% versus 63.6%) and negative predictive value (92.2% versus 92.4%). Furthermore, the false positive and negative rates were comparable for the CE device and permanent pathology. There was a learning curve amongst the surgeons and it was commented by Dixon *et al* [25] that had all images been interpreted appropriately and some re-excised margins identified as CE abnormal in Phase-2 of the study, the rate of re-excision would have been 8% (overall rate of excision 17% for all patients).

## Intelligent knife

An intriguing technology is the ‘intelligent knife’, which analyses the electrosurgical plume of diathermy smoke to determine the structural lipid profile of tissue. This is based on rapid evaporative ionisation mass spectrometry (REIMS) with online chemical analysis of the electrosurgical aerosol. Use of electrical diathermy in the cutting or coagulation mode triggers a cellular explosion from heat dissipation with release of cellular product into a gas phase. Chemical analysis using both cutting and coagulation modality was performed on a normal and tumour breast tissue and validation spectra correlated with tissue microscopic tissue histology. The so-called i-knife has now been optimised for real-time intraoperative analysis of breast tissue and yields high rates of sensitivity and specificity. REIMS shows promising results for detection of positive resection margins intraoperatively (100%) and has a sensitivity of 77.3%. A false positive margin was recorded in only 0.5% of the total dissection time calculated as 69/14,023 spectra [26]. Further validation studies will clarify the performance characteristics and in particular determine the accuracy for intraoperative margin assessment [27].

## Conclusions

Several emergent technologies exist which can potentially improve intraoperative margin assessment and reduce rates of re-excision, but further technological developments are required to augment image processing and facilitate routine clinical usage. Moreover, these will need to compare favourably with direct tissue-based methods, such as frozen section and cytology, which are notably more sensitive and specific than most other methods of margin assessment. Nonetheless, issues such as time, cost and reliability must be factored into any final evaluation and devices such as MarginProbe and ClearEdge may find clinical utility in routine daily practice where surgical workloads are substantial.

## Figures and Tables

**Table 1.** Consensus guidelines and policies for margin mandates.

	Invasive cancer	Pure DCIS
ABS UK*	1 mm	1 mm
SSO-ASTRO**	No tumour at ink	2 mm

* Association of Breast Surgery United Kingdom

** Society of Surgical Oncology and American Society of Radiation Oncology
